# Mucosa Building Bridges: An Uncommon Cause of Colonic Obstruction in Ulcerative Colitis

**DOI:** 10.14309/crj.0000000000002140

**Published:** 2026-05-20

**Authors:** Alexander Siegel, Kyaw Min Tun, Aun Shah

**Affiliations:** 1 Department of Internal Medicine, Creighton University School of Medicine, Omaha, NE

**Keywords:** ulcerative colitis, mucosal bridging, colonic obstruction

## CASE REPORT

**Figure 1.**
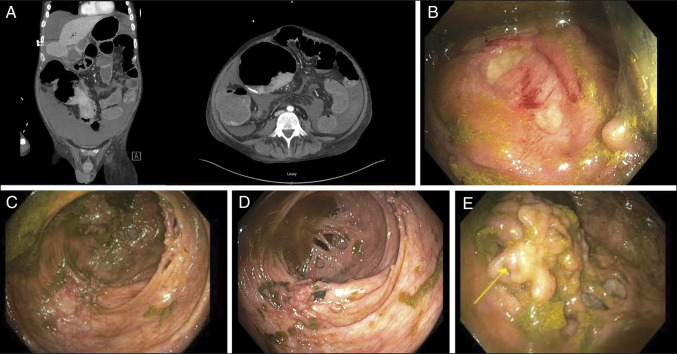
(A) Computed tomography of the abdomen demonstrating diffuse bowel distention with narrowing at the colonic anastomosis. (B) Colonoscopic view of circumfrential inflammatory psuedopolyps. (C) Advancement with a pediatric colonoscope demonstrating multilobulated mucosal projections and extensive mucosal bridging. (D) Cecal examination with additional mucosal bridges forming obstructive intraluminal tracts. (E) Ileocacal valve with pseudopolypoid inflammatory change and adjacent mucosal bridging.

A 50-year-old man with ulcerative colitis (UC), diagnosed in April 2018 after left hemicolectomy for bowel obstruction with pathology confirming UC, presented with a 2-day history of abdominal pain, vomiting, and constipation. He had remained largely untreated since diagnosis. Computed tomography imaging demonstrated distended loops of bowel with a distal colonic mass at the surgical anastomosis (Figure 1). Colonoscopy revealed circumferential pseudopolyps causing luminal narrowing at the anastomosis (Figure 1). Further evaluation with a pediatric colonoscope demonstrated multilobulated mucosal projections and extensive pseudopolyps consistent with mucosal bridging (Figure 1). Cecal examination revealed additional mucosal bridges forming obstructive intraluminal tracts (Figure 1). The ileocecal valve showed pseudopolypoid change with adjacent bridging (Figure 1). A cecal biopsy demonstrated focal cryptitis consistent with UC. Symptoms resolved following endoscopic decompression, and no surgical intervention was required.

Mucosal bridging is an uncommon manifestation of UC.^1,2^ Although fibrotic strictures are uncommon, chronic untreated inflammation can lead to pseudopolyp formation and mucosal remodeling, resulting in bowel obstruction. Previous reported cases often occurred spontaneously and were discovered incidentally. These structural changes, while initially asymptomatic, may predispose patients to obstructive complications if left untreated. Prompt recognition of this complication is essential when guiding management and surveillance strategies in patients with inflammatory bowel disease.^3^

## DISCLOSURES

Author contributions: A. Siegel, KM Tun, and A. Shah contributed to case conception, data collection, image selection, and drafting/revision of the manuscript. A. Siegel is the article guarantor.

Financial disclosure: The authors declare no conflicts of interest.

Previous presentation: Case presented at the American College of Gastroenterology Annual Scientific Meeting in Phoenix, AZ on 10/2025.

Informed consent was obtained for this case report.
